# Water Softening

**Published:** 1911-06-17

**Authors:** 


### Hospital Architecture and Construction.

[Communications on this subject should be marked "Architecture" in the left-hand top corner of the envelope.]

### WATER SOFTENING.

In a series of articles in previous issues—February 25, March 25, and April 8—we dealt with the subjects "Types of Wells," "Creation of Supply," and "Methods of Storage": it now remains to complete the series with the subject of "Water Softening."

Water as it falls in the form of rain is, as before stated, eminently soft, but in its passage through permeable strata by contact with different minerals it becomes "hard"; this makes it undesirable for certain purposes, such as steam boilers and pipes, laundry work, etc. Hardness is expressed in "degrees," each of which is equivalent to one grain of carbonate of lime in each gallon of water. Waters in Great Britain vary in hardness from 1.5 in Aberdeen to 30.0 in Sunderland: London water is about the average with 16 degrees.

The utility of a water-softening plant may be estimated from the fact that the cost of the proper cleaning of a boiler is about 2d. per thousand gallons where the water is of average hardness. To every 1,000 gallons per day evaporated in a boiler it may be assumed that a layer of mud or scale equal to about 150 lb. is deposited each month: this precipitate in its accumulation forms a most effective non-conducting layer between the fire zone and the water, and further clogs the passage-way of the pipes, thus involving a serious extravagance in fuel, while at the same time rendering the purpose of the apparatus more or less ineffective and further seriously damaging its fabric.

The power of a scale deposit to retard heat if of a thickness equal to about one-fourth that of the iron boiler, is equal to the thickness of four extra boiler-plates. For laundry purposes also, hard water has its drawbacks, as it will require a large amount of soap to soften it before the soap can take effect on the clothes, and, further, the scum which hard water gives is detrimental to the clothes and causes black specks which are difficult to remove.

Hardness may be described as of two kinds: temporary and permanent. The former is due to the bicarbonates of lime and magnesia and is so-called because prolonged boiling or evaporation will remove it; the latter is due to sulphates, chlorides, and nitrates of lime and magnesia, and is not diminished by boiling at atmospheric pressure. The means of softening water are as diverse as the different types of water requiring treatment to return them to their original condition as rain-water.

For removing temporary hardness a steam water-heater is often employed. This consists of a number of copper tubes arranged in a wrought-iron casing; live steam passes through the tubes and the water to be softened passes through the spaces between the tubes and the casing. The scale deposits on the tubes and the inside of the casing, and the latter is made to be easily removed so that the scale may be periodically cleaned off. This apparatus may be worked from the boiler exhaust, but is more economical if live steam is the agent employed.

For removing permanent hardness chemicals are added in certain proportions to a water to counteract the hardness it has accumulated in its passage through various soils. There are several methods employed, but the best known are the (*a*) lime process; (*b*) soda process; (*c*) the combined lime and soda process; (*d*) Maignen's process; (*e*) Porter Clark's process. Two tanks are required, one being in use while the hardness is precipitating in the other, and it is usual to have the outlet above the bottom so as not to disturb the precipitate.

About the year 1906 a chemical process of softening, known as the "Permutit," was brought to maturity; it is claimed for this system that it is the only one, other than distillation, which leaves no particle of hardness in the water, and it is reported in the case of a large British firm of woolworkers to have paid for its installation in twelve months by the saving in soap. The softening agent is a manufactured substance known as "Sodium Permutit," and the plants may be installed in dimensions suitable for all establishments, from the smallest cottage hospital to the largest curative colony. This system is one of the most desirable for laundry works.

The "Luminator" system of softening for boiler feeds is a novel one, which consists in physically changing the hard water by running it over corrugated aluminium plates: the physical change results in the calcium and magnesium salts being deposited in the boiler and pipes in the form of soft mud (which can be easily removed by washing or brushing), instead of the hard incrustation or scale, only removable by means of the drill or hammer and chisel. The action being purely physical, no chemicals are required and no attention is necessary beyond the occasional brushing of the aluminium plates to keep them active. This system has proved popular among railway companies.

